# Kinetics of Viral Shedding for Outbreak Surveillance of Emerging Infectious Diseases: Modeling Approach to SARS-CoV-2 Alpha and Omicron Infection

**DOI:** 10.2196/54861

**Published:** 2024-09-19

**Authors:** Ting-Yu Lin, Amy Ming-Fang Yen, Sam Li-Sheng Chen, Chen-Yang Hsu, Chao-Chih Lai, Dih-Ling Luh, Yen-Po Yeh, Tony Hsiu-Hsi Chen

**Affiliations:** 1 Institute of Epidemiology and Preventive Medicine, College of Public Health National Taiwan University Taipei Taiwan; 2 School of Oral Hygiene, College of Oral Medicine Taipei Medical University Taipei Taiwan; 3 Dachung Hospital Miaoli Taiwan; 4 Emergency Department of Taipei City Hospital, Ren-Ai Branch Taipei Taiwan; 5 Department of Public Health, Chung Shan Medical University Taichung Taiwan; 6 Changhua Public Health Bureau Chuang Taiwan

**Keywords:** COVID-19, PCR testing, Ct values, viral load, kinetics of viral shedding, emerging infectious disease, SARS-CoV-2 variants, infection surveillance

## Abstract

**Background:**

Previous studies have highlighted the importance of viral shedding using cycle threshold (Ct) values obtained via reverse transcription polymerase chain reaction to understand the epidemic trajectories of SARS-CoV-2 infections. However, it is rare to elucidate the transition kinetics of Ct values from the asymptomatic or presymptomatic phase to the symptomatic phase before recovery using individual repeated Ct values.

**Objective:**

This study proposes a novel Ct-enshrined compartment model to provide a series of quantitative measures for delineating the full trajectories of the dynamics of viral load from infection until recovery.

**Methods:**

This Ct-enshrined compartment model was constructed by leveraging Ct-classified states within and between presymptomatic and symptomatic compartments before recovery or death among people with infections. A series of recovery indices were developed to assess the net kinetic movement of Ct-up toward and Ct-down off recovery. The model was applied to (1) a small-scale community-acquired Alpha variant outbreak under the “zero-COVID-19” policy without vaccines in May 2021 and (2) a large-scale community-acquired Omicron variant outbreak with high booster vaccination rates following the lifting of the “zero-COVID-19” policy in April 2022 in Taiwan. The model used Bayesian Markov chain Monte Carlo methods with the Metropolis-Hastings algorithm for parameter estimation. Sensitivity analyses were conducted by varying Ct cutoff values to assess the robustness of the model.

**Results:**

The kinetic indicators revealed a marked difference in viral shedding dynamics between the Alpha and Omicron variants. The Alpha variant exhibited slower viral shedding and lower recovery rates, but the Omicron variant demonstrated swifter viral shedding and higher recovery rates. Specifically, the Alpha variant showed gradual Ct-up transitions and moderate recovery rates, yielding a presymptomatic recovery index slightly higher than 1 (1.10), whereas the Omicron variant had remarkable Ct-up transitions and significantly higher asymptomatic recovery rates, resulting in a presymptomatic recovery index much higher than 1 (152.5). Sensitivity analysis confirmed the robustness of the chosen Ct values of 18 and 25 across different recovery phases. Regarding the impact of vaccination, individuals without booster vaccination had a 19% higher presymptomatic incidence rate compared to those with booster vaccination. Breakthrough infections in boosted individuals initially showed similar Ct-up transition rates but higher rates in later stages compared to nonboosted individuals. Overall, booster vaccination improved recovery rates, particularly during the symptomatic phase, although recovery rates for persistent asymptomatic infection were similar regardless of vaccination status once the Ct level exceeded 25.

**Conclusions:**

The study provides new insights into dynamic Ct transitions, with the notable finding that Ct-up transitions toward recovery outpaced Ct-down and symptom-surfacing transitions during the presymptomatic phase. The Ct-up against Ct-down transition varies with variants and vaccination status. The proposed Ct-enshrined compartment model is useful for the surveillance of emerging infectious diseases in the future to prevent community-acquired outbreaks.

## Introduction

In the COVID-19 pandemic, one of the main reasons for the fast spread of SARS-CoV-2 variants was the presymptomatic and persistent asymptomatic infection caused by a group of SARS-CoV-2 mutants, ranging from Wuhan D614G to variants of concern (VOCs) such as Alpha, Beta, Gamma, Delta, and Omicron [1]. Transmission modes, whether before or without the onset of symptoms, are often occult and very hard to identify. To reduce the spread of SARS-CoV-2 and the ascertainment of both presymptomatic and asymptomatic cases, quarantine and isolation with reverse transcription polymerase chain reaction (RT-PCR) tests in the presence or absence of symptoms are applied to suspected infected persons after contact tracing. There are several mathematical models proposed for modeling such an infectious process as susceptible, exposed, infective, and recovering, like the susceptible-exposed-infectious-recovered model and its variants [2-5]. Despite the usefulness of susceptible-exposed-infectious-recovered models and their variants in modeling infectious disease dynamics due to their simplicity, flexibility, and historical success, they have several limitations. These models often assume homogeneous mixing within the population, which may not accurately reflect real-world contact patterns and transmission dynamics. They also use constant transition rates that may not capture the variability and stochastic nature of disease transmission, and they lack detailed individual-level dynamics, particularly in distinguishing between presymptomatic and symptomatic phases and also Ct levels. Our previous studies have also applied a mathematical model to estimate the incidence of presymptomatic and asymptomatic cases and the time required for the transition from the presymptomatic phase to the presence of symptoms. We developed a 4-state stochastic model specifically for data on imported COVID-19 cases, which included parameters like the presymptomatic incidence rate, the median of presymptomatic transmission time to the symptomatic state, and the incidence of asymptomatic cases. This model was applied to empirical data from Taiwan, encompassing various SARS-CoV-2 variants, to bridge the link between the natural infectious properties and different disease phenotypes. The results highlighted the dynamic nature of these parameters across different strains and periods, providing insights into the impact of public health interventions like quarantine and isolation policies [6].

While these models are valuable for providing new insight into the dynamics of strength-based and speed-based infectious processes by SARS-CoV-2 variants, there is a paucity of mathematical models that are proposed for estimating the dynamics of infectious processes associated with the evolution of viral shedding from the time of exposure to infectives to the presence of symptoms. Up to date, there have been few studies so far that have made use of viral load information for surveillance of outbreaks. For example, Hay et al [7] applied the distribution of cross-sectional viral load measured by cycle threshold (Ct) value through RT-PCR to estimate the time-varying reproductive number of SARS-CoV-2 infection and reflect its epidemic trajectory. This is the first study to report the use of a cross-sectional quantitative measure of viral shedding with a Ct value for inferring an epidemic’s trajectory. Recently, Lin et al [8] investigated COVID-19 dynamics across different epidemic waves in Hong Kong, highlighting changes in Ct value distributions across various waves and variants and analyzing the impact of vaccination on clinical outcomes. Furthermore, Puhach et al [9] explored the application of different surveillance methods in inferring epidemic dynamics, particularly using Ct value distributions to estimate epidemic progression. They compared various surveillance methods, including traditional epidemiological data and Ct value-based monitoring, finding that Ct values not only reflect individual viral load changes but can also be used to infer overall epidemic trends and transmission dynamics. These findings indicate that Ct value distributions can serve as a critical tool for epidemic surveillance, helping to timely adjust public health strategies [9]. Dehesh et al [10] also demonstrated that population-level Ct values can predict COVID-19 dynamics, showing a significant negative correlation between average daily Ct values and daily new positive cases, hospitalizations, and deaths, suggesting that Ct values can be a useful indicator for epidemic surveillance.

Regarding vaccination-related studies, Jung et al [11] compared the transmission and viral shedding kinetics of SARS-CoV-2 in vaccinated versus unvaccinated individuals, discovering that fully vaccinated individuals had shorter durations of viable viral shedding and lower secondary transmission rates. Kissler et al [12] analyzed viral dynamics in vaccinated versus unvaccinated persons, showing faster viral clearance in vaccinated individuals and comparing the impacts of different variants. Gowler et al [13] emphasized the importance of viral shedding kinetics and transmission, noting that different vaccination statuses and variants affect the virus's cultivability and transmission patterns, suggesting these factors should be considered in study designs.

These studies underscore the importance of using Ct value dynamics to infer epidemic progression, providing the theoretical foundation for this study. However, there are gaps in the existing research, including the limited focus on variant-specific viral shedding kinetics, insufficient integration of vaccination impact, a lack of comprehensive kinetic models for presymptomatic and symptomatic phases, and the absence of specific indicators to quantify the kinetic movement toward recovery. Our previous studies used a 4-state stochastic model to estimate the incidence of presymptomatic and asymptomatic cases. However, the dynamics of Ct values within and across presymptomatic to symptomatic has not been considered. It is therefore of great interest to address these research gaps by providing a more granular understanding of how different SARS-CoV-2 variants and vaccination statuses influence the kinetics of viral shedding and recovery, offering valuable insights for public health interventions.

The aim of this study is therefore to propose a new Ct-guided compartment model for deciphering the kinetics of viral shedding during the infectious process of 3 compartments (susceptible, presymptomatic, and symptomatic) before recovery or death. A series of useful indicators are also developed to quantify the kinetic movement of viral shedding toward recovery. We applied the proposed Ct-guided model and the developed recovery index to 2 community-acquired outbreaks, including Alpha VOC infection and Omicron VOC infection, with and without the “zero-COVID-19” policy.

## Methods

### Model Specification

**Figure 1:**
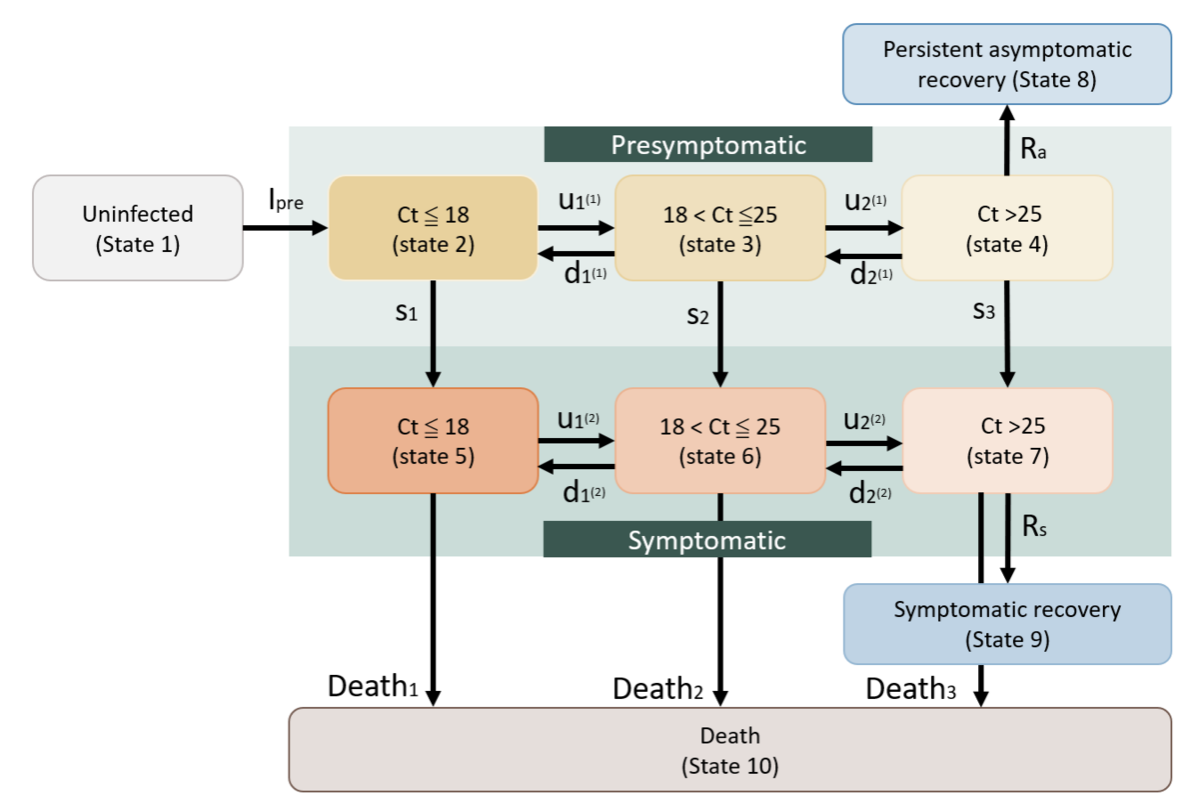
The Ct-guided multistate compartment model for SARS-CoV-2 infection.

Figure 1 shows a specific model for estimating the kinetics of the viral shedding transition within and between the presymptomatic phase and the symptomatic phase before recovery or death for Alpha and Omicron VOCs. Subjects of the underlying susceptible population are defined as an uninfected state (state 1). Once infected, the symptom-based phenotypes of viral shedding during the presymptomatic phase and after the onset of symptoms are expressed by the Ct value with the application of the RT-PCR test to the suspected infected persons. The higher the Ct value, the less concern there is over the transmissibility of SARS-CoV-2 variants. The transition of viral shedding is captured by the change in Ct value in the instantaneous potential (per day). The lower the Ct value, the higher the viral shedding after infection. There are many ways of classifying Ct values into different categories, ranging from 2 to 7 (6 states used in Figure 1). The more refined the Ct value, the closer it is to the continuous property of the Ct value. Different SARS-CoV-2 variants may elicit different cutoffs for the viral shedding transition. Here, we first propose 3 categories of Ct value according to the previous studies, using 18 and 25 as 2 cutoffs for the comparisons of Alpha and Omicron VOCs [14,15]. We performed a sensitivity analysis by changing different cutoffs to assess whether and how the alteration of the cutoff changes the results.

After entering the presymptomatic phase, the transitions between 3 states are determined by 4 transition parameters that govern back and forth movements before entering the symptomatic phase or recovery without showing signs of symptoms, the latter of which would be defined as persistent asymptomatic cases who would be distinct from those presymptomatic cases detected by RT-PCR tests that are often defined as nonpersistent asymptomatic cases. The 4 corresponding transition parameters are also applied to the viral shedding transition within the symptomatic phase before recovery or death. A total of 3 transition parameters are required for bridging the movements from the presymptomatic to the symptomatic phase by 3 categories of viral shedding level. Absorbing parameters included the recovery of persistent asymptomatic cases after departure from the presymptomatic phase, symptomatic cases, and also death.

### The Kinetic Mechanism of Transition Parameters

Following Figure 1**,** there are 3 main compartments, including the uninfected, presymptomatic, and symptomatic, for depicting the entire infectious process on an individual level. The category of Ct level after the RT-PCR test is further incorporated into the presymptomatic phase and the symptomatic phase. It is therefore important to decipher the kinetic mechanism for the transition rates of Ct level within 2 phases after infection and the transition from the presymptomatic compartment to the symptomatic compartment, and also the departure rates for the recovery or death from the 2 main compartments. The first transition parameter from uninfected to presymptomatic infection is called presymptomatic incidence, denoted by I_pre_. The higher the incidence, the higher the transmissibility of SARS-CoV-2. It is postulated that the presymptomatic incidence for Omicron would be higher than that for Alpha. As far as the presymptomatic compartment, the transition parameters governing the underlying kinetic mechanism can be considered as a pair of up-down Ct values within the presymptomatic phase or symptomatic phase and the symptom-surfacing transition from the presymptomatic phase to the symptomatic phase. The first pair of parameters pertaining to the first level of Ct is composed of the Ct-up transition from the lowest level of Ct to the middle level of Ct, defined as the first Ct-up transition rate denoted by u_1_^(1)^, the first Ct-down transition rate denoted by d_1_^(1)^, the opposite of the Ct-up transition, and the first symptom-surfacing transition (s_1_). The same logic would be applied to the second pair of parameters on the second level of Ct, denoted by u_2_^(1)^, d_2_^(1)^, and s_2_. For generalizability, suppose we have m categories of Ct, the jth pair of parameters is determined by u_j_^(1)^, d_j_^(1)^, s_j_ (j=1, 2,…,m–1). The smaller the value of j, the lower the Ct level and the higher the viral load. Regarding the symptomatic compartment model, the pair of transition parameters consist of up-down Ct transitions denoted by u_j_^(2)^ and d_j_^(2)^ and death denoted by death_j_. When reaching the highest level of Ct, there are 2 transition parameters for the highest level of Ct within the presymptomatic compartment, including the departure rate for the recovery as defined as the recovery rate of persistent asymptomatic cases (R_a_) and the symptom-surfacing transition (S_m_). There are only 2 transition parameters: the departure rate for the recovery (R_s_) and the death rate (D). The total parameters required for estimation are generalized to 1+c[2(m–1)]+m+c, where 1 represents the initial compartment, c is the number of infectious compartments, and m is the number of Ct categories. The last 2 elements are related to death and recovery. In Figure 1, c=2 and m=3 are applied.

### Indicators for the Incidence of Infection and the Kinetics of Viral Shedding Toward Recovery

There are 3 indicators proposed here for quantifying the occurrence of infection and the transition of viral shedding leading to recovery. The first indicator for the incidence of infection is the incidence of presymptomatic infection, denoted as I_pre_. The larger the value of I_pre_, the higher the transmissibility and the more frequent contact with infectives.

The second indicator is the recovery index for presymptomatic infection. We proposed the following recovery index for the downstaging of viral load toward the recovery from presymptomatic infection until persistent asymptomatic infection without the manifestation of apparent symptoms, which is expressed by

u_j_^(1)^/(d_j_^(1)^+s_j_) **(1)**

This recovery index is used as the kinetic force toward a lower viral load during the presymptomatic phase. Note that the recovery index is changed to R_a_/(d_m–1_+s_m_) when reaching the status of recovery. The higher the value of this indicator, the faster the recovery would be expected for such persistent asymptomatic cases.

The third indicator is symptomatic infection-recovery or death. The indicator for the kinetic force toward the lower viral load of a symptomatic infection is proposed as follows:

u_j_^(2)^/(d_j_^(2)^+death_j_) **(2)**

Note that the indicator is changed to *R*_s_ when reaching the final destination of recovery.

### Data Collection

**Figure 2:**
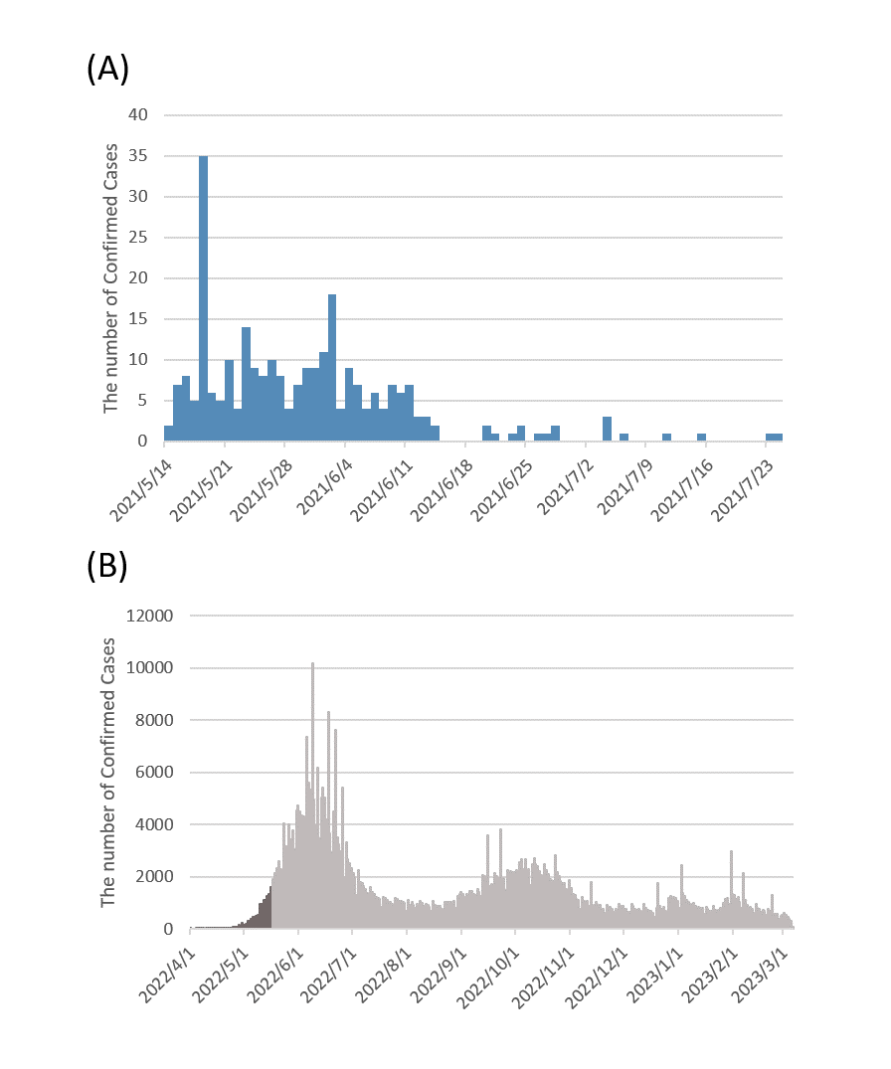
The daily confirmed cases of the 2 outbreaks in Changhua. (A) Alpha. (B) Omicron.

We collected data on 2 epidemic cohorts in Changhua, the middle county of Taiwan, between May 14 and July 24, 2021, dominated by the Alpha variant, and between April 1 and May 16, 2022, dominated by the Omicron variant. Figure 2 shows the 2 epidemic outbreaks. The former was only limited to a small-scale community-acquired outbreak under the “zero-Covid-19” policy with strict nonpharmaceutical interventions (NPI) alert levels but without an available vaccine, whereas the latter resulted in large-scale community-wide outbreaks under the lifting of the “zero-Covid-19” policy with high coverage booster vaccination. Accordingly, the susceptible population related to both collected data is different. The former is limited to people under contact tracing, and the latter involves the entire population at risk. However, in these 2 periods, the Alpha and early Omicron periods before the massive community-acquired outbreak in Taiwan, information on contact tracing through in-person interviews of people with infections, a 14-day quarantine record of close contacts, and repeated measurements of RT-PCR during quarantine and isolation periods for diagnosis, surveillance, and recovery (discharge from hospital if applicable) were available. Data on vaccination status, the date of exposure, symptoms, and repeated measures of viral shedding for each individual were also collected. Table 1 shows the descriptive data on the distribution of Ct for both the Alpha VOC and Omicron VOC.

**Figure 3:**
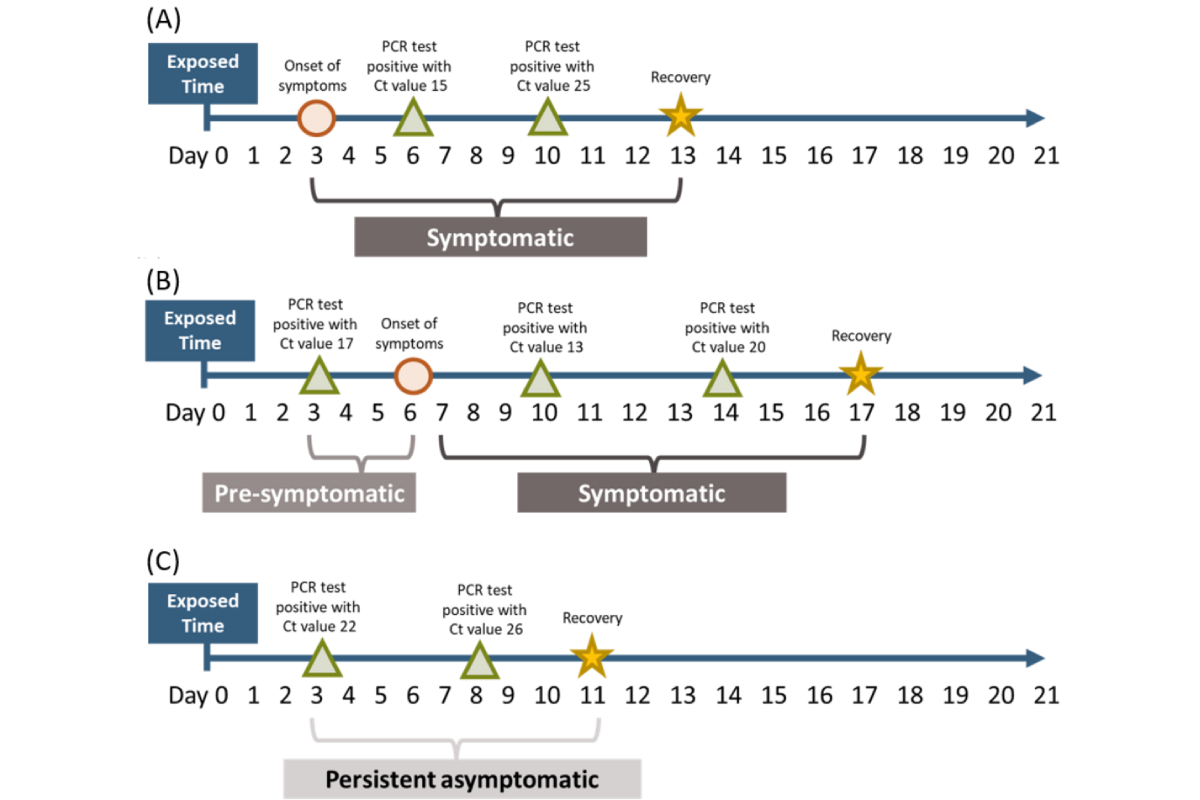
Data realizations of 3 hypothetical cases corresponding to 3 different detection modes.

Accordingly, we are able to distinguish 3 types of data modes, including symptomatic, presymptomatic, and asymptomatic cases upon RT-PCR diagnosis, in light of the abovementioned model (state 2-state 9) based on information on the timeline of exposure, quarantine, symptoms, and diagnosis. Figure 3 and Textbox 1 show 3 hypothetical cases for the corresponding scenarios.

**Table 1:** Presymptomatic, asymptomatic, and symptomatic cases and their distributions of RT-PCR repeated measurements for Alpha VOC and Omicron VOC infections.

		Alpha	Omicron
Population, n		—^a^	1,288,561
Contact tracing, n		8018	—
Infection, n		269	1118
Asymptomatic, n, %		24 (8.9%)	291 (26%)
Symptomatic, n, %		245 (91.1%)	827 (73.9%)
Repeated measurement of RT-PCR, n, %		963	1306
**Asymptomatic or presymptomatic**			
	Total, n	109	335
	<18, n, %	23 (21.1%)	40 (11.9%)
	18-25, n, %	26 (23.9%)	216 (64.5%)
	>25, n, %	60 (55.0%)	79 (23.6%)
**Symptomatic**			
	Total, n	854	971
	<18, n, %	165 (19.3%)	150 (15.4%)
	18-25, n, %	177 (20.7%)	673 (69.3%)
	>25, n, %	512 (60.0%)	148 (15.2%)

^a^Not applicable.

**Hypothetical cases for different scenarios.**

- The symptomatic case (Figure 3A) refers to one diagnosed (first RT-PCR positive result) after the onset of symptoms. This case went through an unobserved presymptomatic phase (state 2-state 4), stayed in state 5 at day 6 and state 7 at day 10, and moved to state 9 at day 13.
- A presymptomatic case showed no symptoms before the first positive RT-PCR test but developed symptoms afterwards (Figure 3B). The trajectory of this case was state 2 at day 3, states 5 and 6 at days 10 and 14, respectively, and finally moved to state 9 at day 17.
- A persistent asymptomatic case again had no symptoms before the first positive RT-PCR test and never had any symptoms until the end of the quarantine period (Figure 3C). This case first showed up in state 3 at day 3, stayed in state 4 at day 8, and reached recovery in state 8 at day 11.

### Multistate Markov Model and Bayesian Markov Chain Monte Carlo for Parameter Estimation

We developed a ten-state multistate Markov model for fitting the proposed model in Figure 1 to the empirical data on Ct repeated measurements, as mentioned earlier. The details of defining transition probabilities between ten states and the development of likelihood functions and Bayesian posterior distributions are derived and given in Multimedia Appendix 1. All the parameters encoded in Figure 1 and the intensity matrix in equation (1) of Multimedia Appendix 1 were estimated using the Bayesian Markov chain Monte Carlo method with the Metropolis-Hastings sampling algorithm for simulating the approximate samples based on the posterior distribution, which was derived from the prior information on all relevant parameters in combination with the likelihood function formed by the empirical data with transition probabilities between Ct-enshrined up-down transitions and recovery or death. The details are given in Multimedia Appendix 1.

### Ethical Considerations

The study was approved by the Institutional Review Board of Taipei Medical University (TMU-JIRB: N202007018) for the authors to have permission to use the original data. The provision of individual information, including health data, travel history, occupation, contact history, and cluster information was mandatory during the outbreak period as justified by the Taiwan Communicable Disease Control Act. According to the act, consent for the retrieval of individual information related to the containment of EID outbreaks can be waived under government auspices.

## Results

### Estimated Transition Parameters

Table 2 shows the detailed estimates of transition parameters governing the kinetics of viral shedding within and between 2 (presymptomatic and symptomatic) compartments of Alpha and Omicron infection. As far as the first susceptible compartment, the expected presymptomatic incident cases per day for Alpha VOC cluster infection were 326 (8018×0.047) among 8018 contact tracing persons and 2319 (1,288,561×0.0018) for the county-wide spread of the Omicron VOC among 1,288,561 residents.

**Table 2:** Estimated daily transition rates by variants and vaccination status.

	Alpha	Omicron		
		Overall	Unvaccinated	Vaccinated
Presymptomatic incidence (I_pre_)	0.0407	0.0018	0.0019	0.0016
Presymptomatic first Ct-up transition rate (u_1_^(1)^)	0.1693	0.2905	0.2988	0.2613
First symptom-surfacing transition (s_1_)	0.2693	0.6152	0.6397	0.5850
Presymptomatic first Ct-down transition rate (d_1_^(1)^)	0.1186	0.0239	0.0350	0.0052
Presymptomatic second Ct-up transition rate (u_2_^(1)^)	0.2041	0.2803	0.2585	0.3000
Second symptom-surfacing transition (s_2_)	0.1266	0.0523	0.0399	0.0399
Presymptomatic second Ct-down transition rate (d_2_^(1)^)	0.0159	0.0250	0.0427	0.0280
Third symptom-surfacing transition (s_3_)	0.0482	0.0006	0.0011	0.0011
Recovery rate of persistent asymptomatic cases (R_a_)	0.0512	0.0957	0.1055	0.1008
Symptomatic first Ct-up transition rate (u_1_^(2)^)	0.2229	0.3033	0.4193	0.2424
Symptomatic first Ct-down transition rate (d_1_^(2)^)	0.0273	0.0756	0.1877	0.0243
Symptomatic second Ct-up transition rate (u_2_^(2)^)	0.2780	0.2767	0.2651	0.2954
Symptomatic second Ct-down transition rate (d_2_^(2)^)	0.0279	0.0164	0.0210	0.0120
Departure rate for the recovery (R_s_)	0.0705	0.1431	0.1534	0.1365
Death_1_	0.0013	—	—	—
Death_2_	0.0046	—	—	—
Death_3_	0.0026	—	—	—

^a^Negligible.

**Figure 4:**
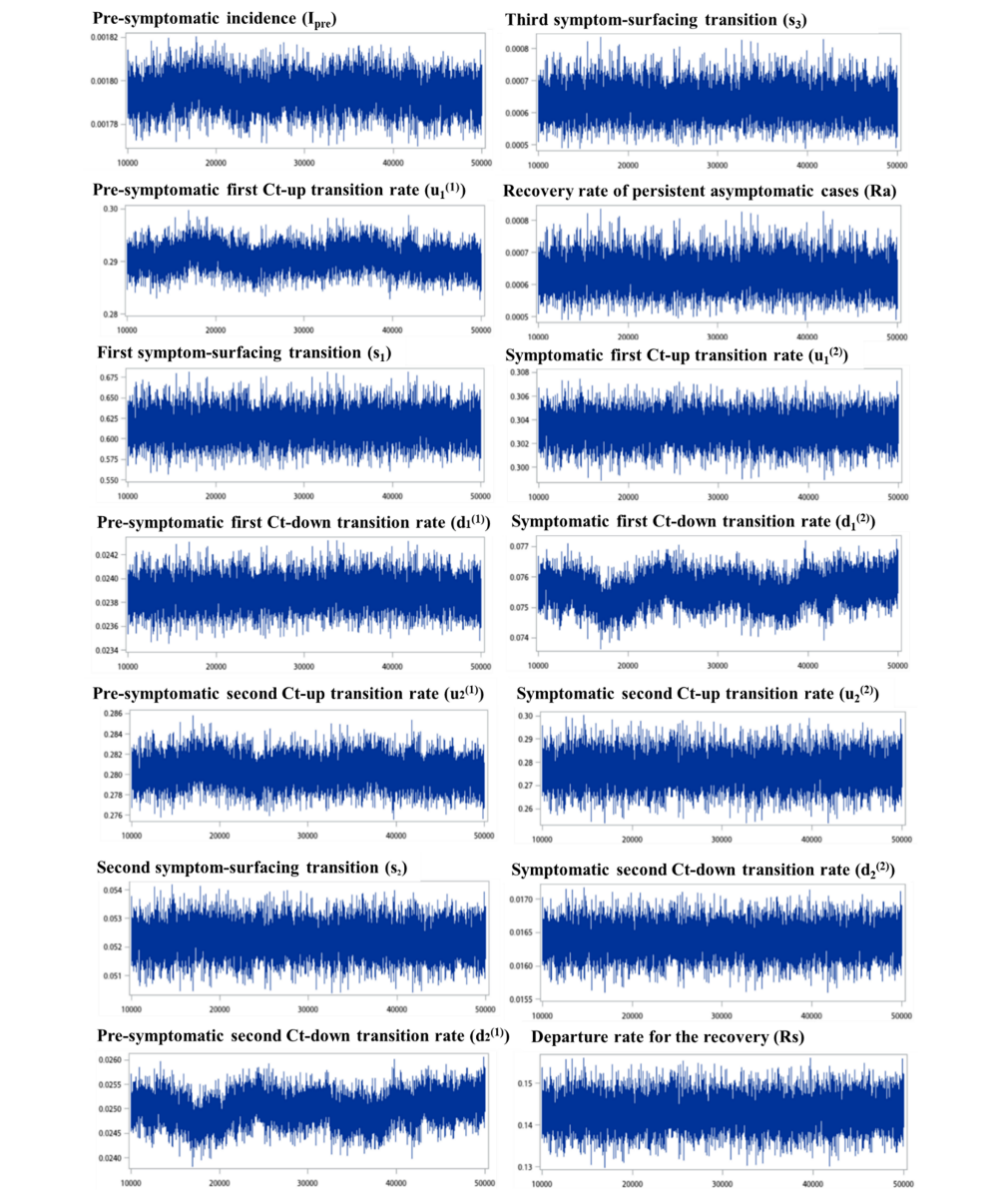
Metropolis-Hastings approximate samples for the estimated parameters of posterior distributions.

Figure 4 shows the approximate Bayesian Markov chain Monte Carlo Metropolis-Hastings samples for the estimated transition parameters, as shown in Table 2. In assessing the convergence of the estimated parameters, we used the standard diagnostics, as illustrated in Figure 4, which presents the Metropolis-Hastings approximate samples for the estimated parameters of all posterior distributions. The trace plots demonstrate stability, consistency, and low autocorrelation, indicating that the samples reach a stationary distribution and that the chains are mixing adequately. The results show very good convergence of the estimated parameters.

### Kinetics of the Ct-Up-Down Infectious Process of Alpha VOC

Table 3 shows the kinetics toward the reduction of viral load with the proposed indicator in light of the estimated transition rates shown in Table 2 for Alpha VOC. The kinetic indicator for the Ct-up transition to reduce viral load increased from 0.44 (0.17/(0.27+0.12)) for the first de-escalation to 1.49 (0.20/(0.13+0.016)) for the second de-escalation. The final recovery rate against the symptom-surfacing transition for an asymptomatic infection was 1.10 (0.051/0.048).

The bottom panel of Table 3 shows that once surfacing to symptoms, the Ct-up transition increased from 8.71 (0.22/0.03) at the first de-escalation to 10.15 (0.28/0.027) at the second de-escalation of Ct. The final recovery rate was 0.07 per day. Note that the death rate was smaller compared with the recovery rate.

**Table 3:** Estimated recovery index by variants and vaccination status.

Recovery index		Alpha	Omicron		
			Overall	Unvaccinated	Vaccinated
**Presymptomatic-asymptomatic infection-recovery**					
	Presymptomatic recovery index 1: u_1_^(1)^/(s_1_+d_1_^(1)^)	0.44 (0.31-0.52)	0.45 (0.44-0.47)	0.44 (0.41-0.48)	0.44 (0.42-0.46)
	Presymptomatic recovery index 2: u_2_^(1)^/(s_2_+d_2_^(1)^)	1.49 (0.88-2.28)	3.63 (3.53-3.73)	3.14 (2.86-3.41)	4.42 (4.27-4.57)
	Presymptomatic recovery index 3: R_a_/s_3_	1.10 (0.61-1.65)	152.5 (129.6,177.2)	97.81 (84.4-111.3)	93.5 (79.13-107.3)
**Symptomatic infection-recovery or death**					
	Symptomatic recovery index 1: u_1_^(2)^/(d_1_^(2)^+death_1_)	8.71 (4.94-13.64)	4.01 (3.99-4.04)	2.24 (2.00-2.46)	10.25 (7.08-13.67)
	Symptomatic recovery index 2: u_2_^(1)^/(d_2_^(2)^+death_2_)	10.15 (7.93-13.04)	16.88 (16.6-17.2)	12.74 (10.57-15.03)	24.79 (21.18-28.65)
	Symptomatic recovery index 3: R_s_	0.07 (0.06-0.08)	0.14 (0.14-0.15)	0.15 (0.15-0.16)	0.14 (0.14-0.14)

### Kinetics of the Ct-Up-Down Infectious Process of Omicron VOC

Regarding the Omicron infection, Table 3 shows the kinetic indicator for the Ct-up transition to reduce viral load increased from 0.45 (0.29/(0.62+0.02)) for the first de-escalation to 3.63 (0.28/(0.05+0.03)) for the second de-escalation. The final recovery rate for asymptomatic infection against the symptom-surfacing transition was 152.5 (0.0957/0.0006).

The bottom panel of Table 3 shows that once symptoms surfaced, the Ct-up transition increased from 4.01 (0.30/0.076) at the first de-escalation to 16.88 (0.28/0.016) at the second de-escalation of Ct. The final recovery rate per day was 0.14. Compared with the recovery rate, the death rate was almost negligible.

### Kinetics of the Ct-Up-Down Infectious Process by Vaccination for Omicron

Table 3 also shows each transition parameter by Alpha and Omicron infection, classified by booster vaccination or no vaccination. It is interesting to note that the presymptomatic incidence rate among persons without booster vaccination was higher by 19% than among persons with booster vaccination. Those with booster vaccination who once had vaccine breakthrough infection had identical Ct-up transition at first de-escalation compared with those in the absence of booster vaccination, whereas the kinetics of Ct-up transition at second de-escalation were higher among those with booster vaccination than those in the absence of booster vaccination. The estimated Ct-up transition rates were higher for those with booster vaccination compared with those without booster vaccination when reaching the symptomatic phase. However, once the Ct level exceeded 25, there was a similar higher recovery rate for persistent asymptomatic infection and also similar recovery rates after the onset of symptoms, regardless of booster vaccination.

### Ct-Guided Kinetics Curves of Alpha and Omicron Infectious Processes

**Figure 5:**
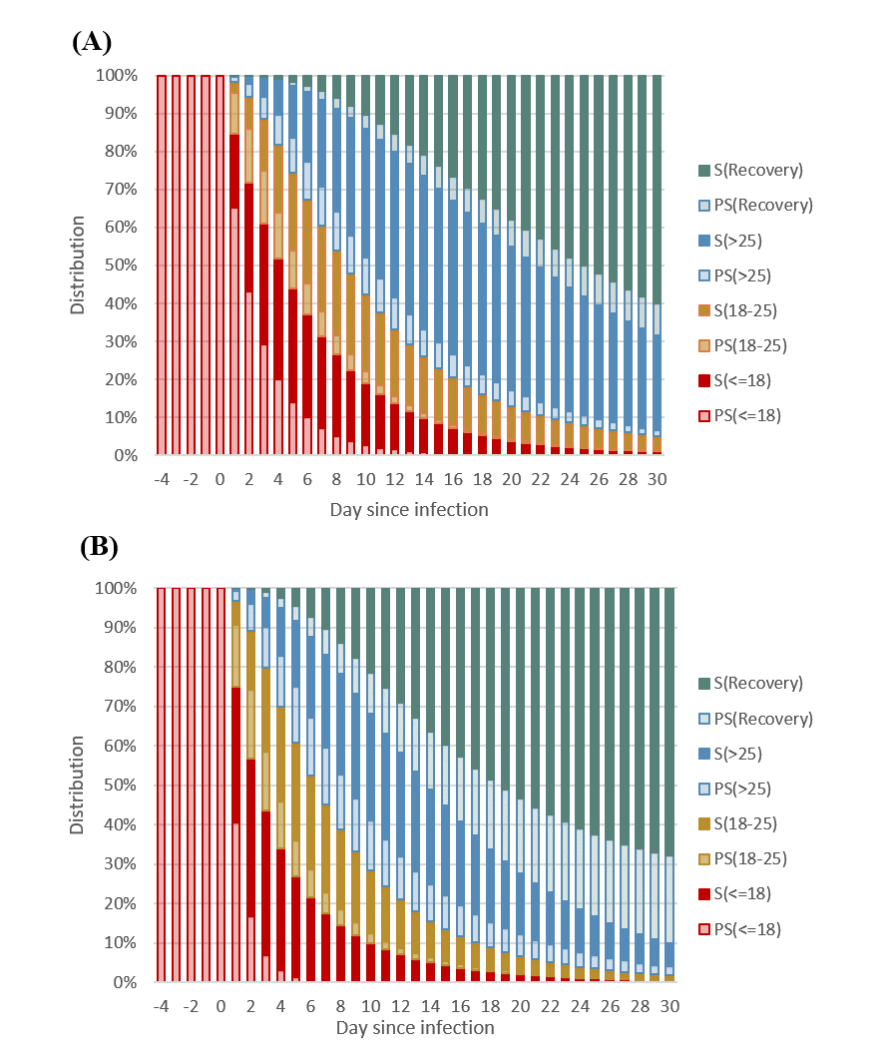
Dynamic curves since infection. (A) Alpha. (B) Omicron. PS: presymptomatic; S: symptomatic.

Based on these estimated transition parameters for both Alpha and Omicron VOCs, a series of dynamic presymptomatic (in light color) and symptomatic curves (in deep color) from days of infection (–4 days), through onset of symptoms (day 0), until recovery or death during 1 month are presented in Figure 5. Once infected, the viral shedding manifested from the high (Ct<18 in red), medium (18-25 Ct in brown), and low (>25 Ct in blue) levels, and 2 recovery modes, persistent asymptomatic recovery (in light green) and symptomatic recovery (in deep green). The dynamic of viral shedding was faster for Omicron VOC infection than Alpha VOC infection because of the following findings. The Omicron infection had a higher proportion of persistent asymptomatic recovery (light green) compared with the Alpha infection. In contrast, the low viral load level in the symptomatic phase (deep blue) was higher for the Alpha infection than that for the Omicron infection. Both results were attributed to the faster de-escalation from high viral load level to medium and low viral load level before surfacing to the symptomatic phase for Omicron infection (see red and brown curves), which were consistent with the findings from Table 2.

### Sensitivity Analysis for Lower and Upper Cutoff of Ct

**Figure 6:**
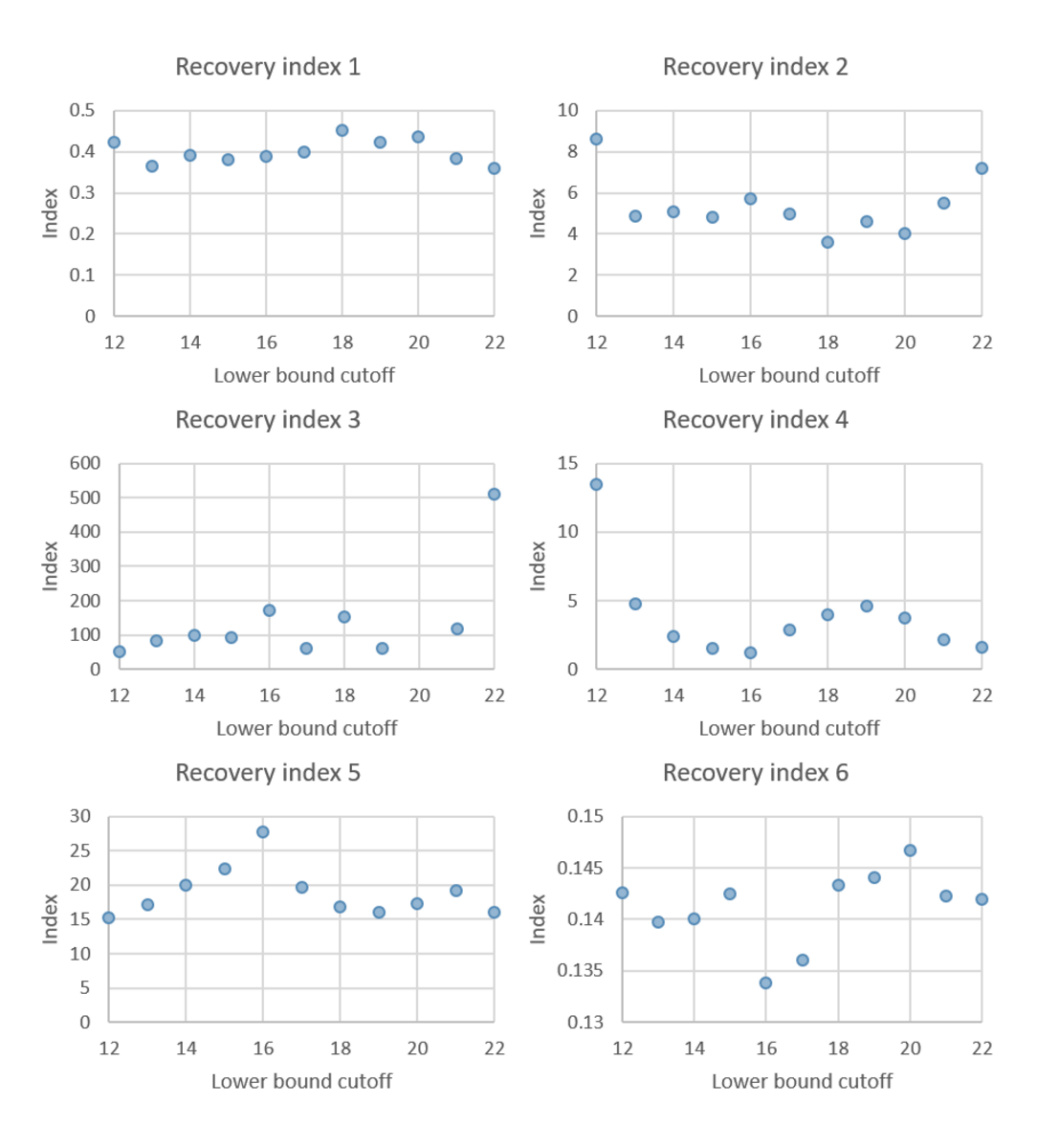
Sensitivity analysis of the lower bound cutoff for the Ct-guided multistate compartment model.

**Figure 7:**
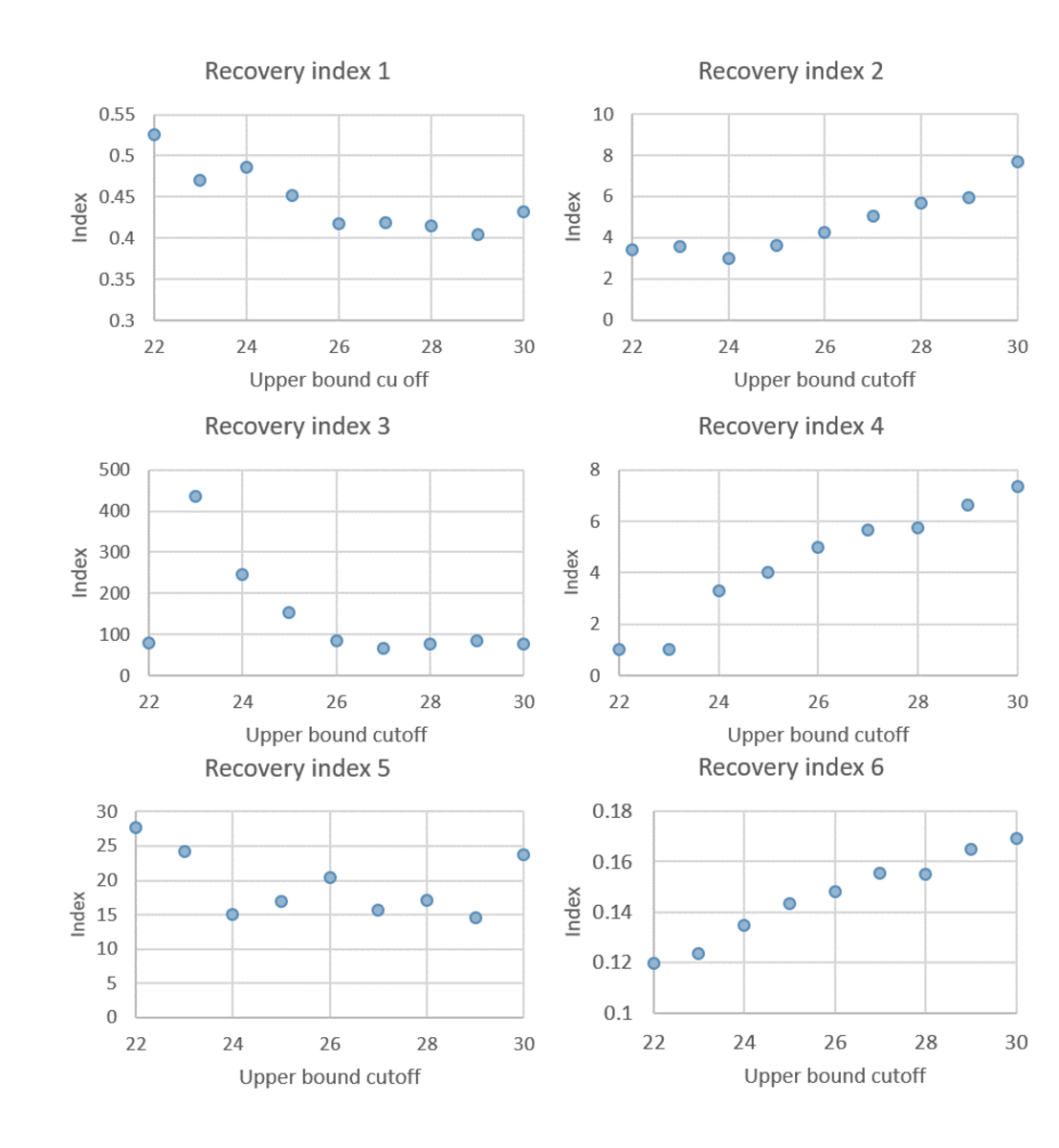
Sensitivity analysis of the upper bound cutoff for the Ct-guided multistate compartment model.

Figures 6 and 7 show the sensitivity analysis of changing the cutoffs of the lower and upper bounds of Ct-defined states for up-down transitions in Figure 1. The results are robust between 14 and 20 Ct values for the lower bounds and greater than 25 Ct values for the upper bound for both presymptomatic recovery and the symptomatic index. Both findings lend support to our choice of 18 and 25 Ct values used in Figure 1.

## Discussion

### Principal Results

This is the first study to elucidate the kinetics of up-down viral shedding enshrined within and between presymptomatic and symptomatic compartments in the light of Alpha and Omicron infections. The proposed recovery indices are very useful for the surveillance of the force toward the reduction (de-escalation) of viral load and further toward the state of recovery when facing emerging infectious diseases in the future. The effect size of multiple recovery indices would reflect intrinsic characteristics of effective reproductive number in terms of contact rate, transmissibility, and infectiousness period and would also be determined by containment measures (including NPIs, anti-viral therapy, and vaccination) for various emerging variants such as SARS-CoV-2 and different containment measures and scales of community-acquired outbreaks. With the current data on the Taiwan scenario, vaccines had not yet been available for administration and were implemented with very strict NPIs by Alert Level 3 during the COVID-19 Alpha VOC period. During the COVID-19 Omicron BA.2 period, primary booster vaccination and booster vaccination had already reached at least 70%, although Omicron infections are not fully prevented by the uptake of the vaccine. However, the proposed model for the surveillance of the kinetics of up-down viral shedding can be flexibly accommodated to various scenarios of emerging infectious disease.

The usefulness of the proposed surveillance indicators is therefore 2-fold. First, it is very helpful for elucidating the dynamics of 2 pathways leading to recovery: one is related to the recovery of persistent asymptomatic cases (state 8), and the other is related to symptomatic recovery (state 9). From Figure 1, the underlying mechanism for the pathway leading to the recovery of persistent asymptomatic cases is mainly determined by 3 recovery indices with the findings shown in Table 2. When the presymptomatic recovery index is greater than 1, the force of moving toward recovery is likely. The larger the presymptomatic recovery index, the faster the recovery. The larger presymptomatic recovery index would imply a higher possibility of precluding presymptomatic transmission from further widespread community-acquired outbreaks. Whether the magnitude of the index is greater than 1 is determined by whether the Ct-up transition rate is larger than the 2 opposing rates of competing transition, the Ct-down transition, and the first symptom-surfacing rate.

In the current Taiwan scenario, the first recovery index, the first de-escalation of a high viral load level (Ct18), for both Alpha and Omicron infections is smaller than 1, as the first Ct-up transition rate was lower than the sum of 2 opposing forces, the first symptom-surfacing rate and the first Ct-down transition rate. Most importantly, the second recovery index, the second de-escalation of the medium viral load level (Ct 25-18), for both Alpha and Omicron is larger than 1 as the second Ct-up transition rate was larger than 2 opposite forces, the second Ct-down transition rate and the second symptom-surfacing rate. The third recovery index for both variants is also larger than 1. More importantly, the second recovery index, the de-escalation of the medial viral load level, plays a crucial intermediate role in facilitating persistent asymptomatic recovery. It is very interesting to note that both Alpha and Omicron VOC infections have the same first recovery index that is smaller than 1, whereas the second recovery index and, particularly, the third recovery index are larger for the Omicron VOC compared with the Alpha VOC. These findings suggest that as both second and third recovery indices are larger for Omicron compared with Alpha during the presymptomatic phase, the odds of persistent asymptomatic cases for the Omicron VOC were 138-fold higher compared with the Alpha VOC. The remarkable contrast of the second and third recovery indexes between 2 variants also reveals the susceptible-infective-recovery process in commensuration with the “zero-COVID-19 policy during the Alpha VOC pandemic and with the lack of a “zero-COVID-19” policy during the Omicron pandemic.

Information provided from the symptomatic recovery index may represent transmissibility and the duration of infectiousness of the symptomatic cases; the higher the symptomatic recovery index, the lower the transmissibility and the shorter the period of infectiousness during hospitalization, which would bring the effective reproductive number down to less than 1 sooner following a community-acquired outbreak. However, the effect size of symptomatic recovery indices may not be comparable between variants or subvariants, although the findings indicate that all of them are larger than 1. This is mainly because the containment measures of various variants or subvariants may vary from place to place. In our scenario, when the outbreak of Alpha VOC infection occurred, vaccines were not available, and only NPIs like isolation together with hospitalization following the “zero-COVID-19” policy could be strictly controlled and provided. The strict policy of isolation prompts symptomatic recovery indices to become larger for faster recovery. This also accounts for why community-wide Alpha epidemic outbreaks could be controlled in Taiwan at that time. However, it should be noted that the symptomatic recovery index may not be as large as noted here when the proposed Ct-up-down-transition model is applied to other settings with a community-wide epidemic outbreak.

During the Omicron BA.2 period, “zero-COVID-19” has been gradually lifted, and the main contrast between the 2 variants is mainly manifested in the presymptomatic recovery index, as mentioned earlier. The main highlight during the Omicron pandemic period is the vaccination. Those with booster vaccination during the Omicron BA.2 period had larger recovery indices compared with those without booster vaccination. Therefore, the second usefulness of the proposed surveillance indicators is that those recovery indices can be used for decoding the effectiveness of booster vaccinations associated with Ct-up-down transitions and recovery or death. In our Omicron scenario, booster vaccination brings down 29% for presymptomatic recovery index 2, 78% for symptomatic recovery index 1, and 49% for symptomatic recovery index 2, but it does not affect presymptomatic recovery index 1 and 3 as well as symptomatic recovery index 3. Such findings provide a new insight into why and how booster vaccination can make a greater contribution to moderate-to-severe COVID-19 cases than recovery, as demonstrated in several studies of the effectiveness of booster vaccination [16,17].

### Comparison With Prior Literature

Our study provides a detailed examination of the kinetics of viral shedding for both Alpha and Omicron variants, introducing recovery indices to quantify the de-escalation of viral load toward recovery. Previous research has emphasized the importance of Ct values in monitoring and predicting epidemic dynamics. For example, Hay et al [7], Puhach et al [9], and Dehesh et al [10] demonstrated the utility of cross-sectional viral load distributions measured by Ct values to estimate reproductive numbers and reflect epidemic trajectories. These studies effectively highlighted the use of Ct values for epidemic monitoring and showed significant correlations between average daily Ct values and new cases, hospitalizations, and deaths [7,9,10].

However, while these studies provided valuable insights into general epidemic trends and transmission dynamics, they did not differentiate between the presymptomatic and symptomatic phases of viral shedding, nor did they provide detailed kinetic models that capture the transition within and between these phases. Our study addresses these gaps by offering a comprehensive kinetic model for not only distinguishing the presymptomatic phase from the symptomatic phase but also eliciting specific recovery indices to monitor the effectiveness of containment measures and vaccination. This allows for a more granular understanding of how viral load dynamics evolve over the trajectory of infection and recovery, providing practical tools for epidemic monitoring and intervention evaluation.

In terms of vaccination impact, studies by Jung et al [11], Kissler et al [12], and Gowler et al [13] focused on comparing viral shedding kinetics between vaccinated and unvaccinated individuals, highlighting shorter durations of viable viral shedding and faster viral clearance in vaccinated individuals. These observations are consistent with our findings, which also demonstrate that vaccinated individuals, particularly those who received booster doses, exhibit shorter durations of viral shedding and faster recovery rates. These studies emphasized the importance of considering vaccination status and variant differences but did not integrate these observations into a kinetic model for considering different infection stages and specific recovery trajectories. Our study extends their methodology by incorporating vaccination status into our kinetic framework, thereby providing insights into how vaccination influences the entire infectious process, including the likelihood of recovery and reduction in viral load [11-13].

### Limitation

The major limitation of this study is that we do not have the opportunity to apply the proposed Ct-up-down transition model to external data. However, as mentioned earlier, the empirical findings are highly dependent on the real-world data pertaining to the strain of the new emerging infectious disease and also the policy of containment measures and the coverage of vaccination. Therefore, the external validation of the proposed model is impracticable and can only be subjected to real-world data applications. It is therefore suggested that the simulated kinetic curves with different combinations of the estimated parameters for new emerging infectious diseases as shown in Figure 4 using artificial intelligence and machine learning are highly recommended to project the trajectory of kinetic viral shedding.

### Conclusions

In conclusion, the proposed Ct-enshrined compartment model effectively deciphers the kinetics of viral shedding, making it a valuable tool for the surveillance of emerging infectious diseases. By analyzing both Alpha and Omicron variants, the model highlights significant differences in viral shedding dynamics, with Omicron exhibiting faster viral shedding and higher recovery rates compared to Alpha.

Additionally, the model demonstrates the impact of vaccination, showing that vaccinated individuals, especially those with booster doses, experience shorter viral shedding durations and quicker recovery. These findings align with previous studies and underscore the importance of incorporating vaccination status into kinetic models to better understand and manage infectious disease outbreaks.

The flexibility of the model allows it to be adapted to various scenarios, enhancing its utility in real-time epidemic surveillance and intervention evaluation. Overall, this study provides a robust framework for tracking viral load dynamics and assessing the effectiveness of public health measures.

## Appendix

Multimedia Appendix 1 Supplementary information on model specification.

## Abbreviations

Ct: cycle threshold
NPI: nonpharmaceutical intervention
RT-PCR: reverse transcription polymerase chain reaction
VOC: variant of concern

## Data Availability

Aggregated data that support the findings of this study are available upon request from the corresponding author (THHC). The original data sets used in this study are not publicly available due to regulations but are available from the corresponding author on reasonable request.
