# Supplementary material for: Kinetics of Viral Shedding for Outbreak Surveillance of Emerging Infectious Diseases: Modeling Approach to SARS-CoV-2 Alpha and Omicron Infection
Source: JMIR Public Health Surveill. 2024 Sep 19;10:e54861. doi: 10.2196/54861 (PMC11450350; doi:10.2196/54861)
Supplement: Multimedia Appendix 1 [file publichealth_v10i1e54861_app1.docx]

**Supplementary**

***Model specification***

To capture the evolution of COVID-19 disease through with the consideration of both viral shedding and clinical symptoms, we developed a discrete-state and continuous-time stochastic process with. **Figure 1** shows the structure of the proposed multistate model. Subjects were in the normal state (state 1) corresponding to the susceptible states before contact and been infected by infectious cases. States regarding viral shedding can be defined in a generalized approach. Without the loss of generality, the evolution of COVID-19 in terms of viral shedding after being infected, was categorized into three levels by using the level of Ct value, ranging from low (Ct>25), medium (18< Ct <=25), and high (Ct<=18). With the evolution of COVID-19 disease, the level of viral shedding can progress or regress. For each level of viral shedding, the COVID-19 case can present without clinical symptom (state 2 (high viral shedding), state 3 (medium viral shedding), and state 4 (low viral shedding)) or with clinical symptom (state 5 (high viral shedding), state 6 (medium viral shedding), and state 7 (low viral shedding)). While COVID-19 cases can recover after the clearance of viral shedding for subjects with (state 9) or without (state 8) clinical symptom, patients with clinical symptom in state 5, 6 and 7 are at risk of death (state 10).

The transition intensity matrix (***Q***) corresponding to the continuous time ten-state Markov process of Figure 1 can be specified by (1).

$\mathbf{Q}=$


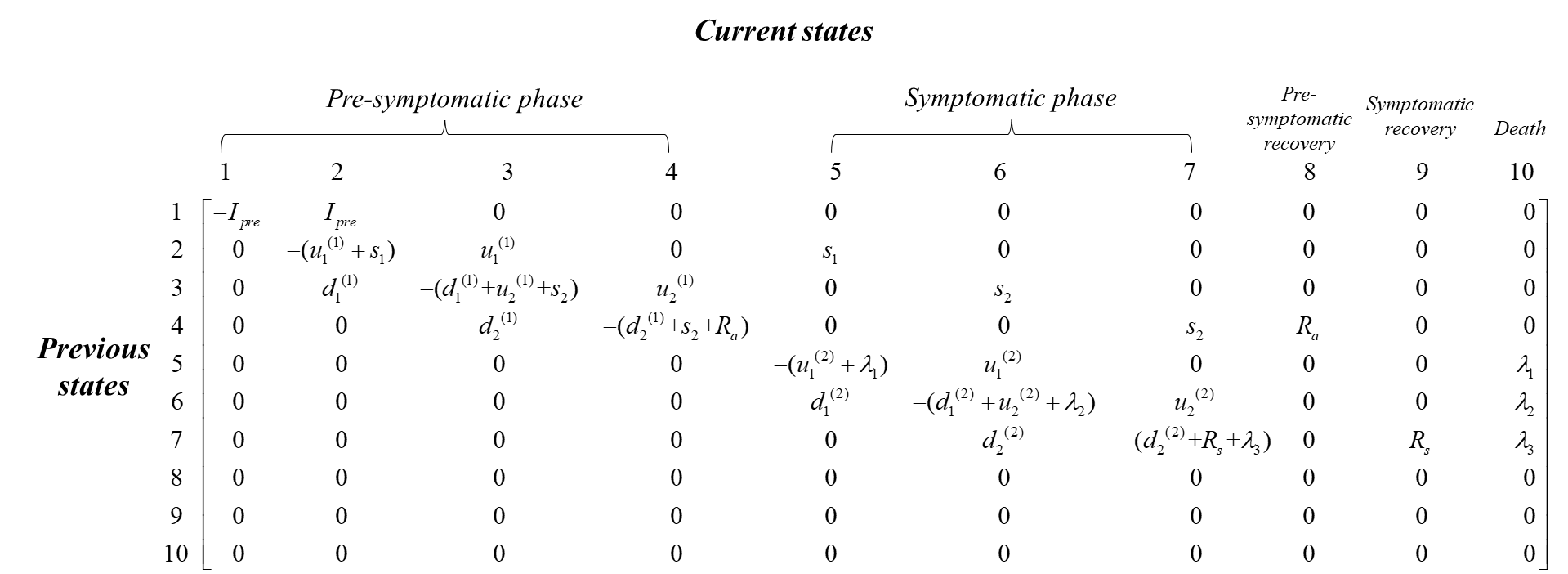


(1)

The corresponding transition probability matrix, $\boldsymbol{P}\left( t \right)$given the period *t* for COVID-19 evolution can be derived from the backward Kolmogorov equation, subject to $\boldsymbol{P}\left( 0 \right)=\boldsymbol{I}$ and by using the spectral analysis,

$\boldsymbol{P}\left( t \right)=\boldsymbol{A}^{\boldsymbol{-1}}diag(e^{I_{pre}t}$,$e^{u_{1}^{(1)}t}$,$e^{u_{1}^{(2)}t},e^{u_{2}^{(1)}t}$,$e^{d_{2}^{(2)}t},e^{d_{1}^{(1)}t}$,$e^{d_{1}^{(2)}t},e^{d_{2}^{(1)}t}$,$e^{d_{2}^{(2)}t},e^{R_{a}t}, e^{R_{s}t},e^{\lambda_{1}t}, e^{\lambda_{2}t},e^{\lambda_{3}t}$) **A** (2)

where $I_{pre}, u_{1}^{(1)}, \ldots, \lambda_{3}$are eigenvalues of intensity matrix (***Q***) and ***A^-1^*** and ***A*** are the right and left eigenvectors of ***Q*** matrix.

***Likelihood function***

Let random variable X(t) denote the observed state of COVID-19 regarding the states of viral shedding and clinical symptom as mentioned above at time t, X(t) $\in$ Ω state space defined in **Figure 1**, Ω = {1, 2, …, 10}. After the realization of X(t), for a total of subjects with serial observation on the defined COVID-19 states, the likelihood function can be formed by using the transition probability matrix as follows,

$L\left( y | \boldsymbol{u,d,}R_{a},R_{s},\lambda_{1},\lambda_{2},\lambda_{3} \right)=\prod_{v=1}^{n_{ij}} \frac{dP_{ij}\left( t_{v} \right)}{dt}$ (3)

where$n_{ij}$represents the number of observed transition modes across the COVID-19 states from $i$ to $j$. By combining the likelihood function of (3) with the non-informative prior distributions proposed as follows,

$\pi(\theta)\sim$ Gamma (0.01, 10000)

$\theta$={$\boldsymbol{u,d,}R_{a},R_{s},\lambda_{1},\lambda_{2},\lambda_{3}$}

the posterior distribution was therefore derived. Approximate metro-polis hasting samples for estimating the parameters encoded in the posterior are also demonstrated in **Figure 4** with good convergence for getting consistent parameters.
